# AN INNOVATIVE PLATFORM BASED ON WEARABLE SENSOR TO QUANTIFY FRAILTY PHENOTYPES

**DOI:** 10.1093/geroni/igz038.2523

**Published:** 2019-11-08

**Authors:** He Zhou, Bijan Najafi

**Affiliations:** Baylor College of Medicine, Houston, Texas, United States

## Abstract

This study evaluated an innovative wearable sensor based platform (instrumented trail-making task, iTMT) to quickly quantify frailty phenotypes, without the need of walking test. 61 older adults (age=72.8 ± 9.9years, BMI=27.4±4.9kg/m2) were recruited and assessed by Fried Frailty Criteria to determine frailty phenotypes. All subjects participated the iTMT test by standing in front of a computer, wearing a wearable sensor on the front lower shin. The sensor tracked the subject’s ankle movement and projected it on a computer-screen as a moving cursor at real-time. The subject rotated the ankle joint to navigate the cursor to reach 5 indexed circles (including numbers 1-to-3 and letters A&B placed in random orders) in the alternative order of numbers and letters. The iTMT required coordination of brain and body movement, testing subject’s cognitive-motor function. The sensor quantified ankle-rotation biomechanics during the test. All subjects completed the iTMT with average time less than 3 minutes. The ankle-rotation velocity generated during the test distinguished between the presence and absence of Slowness phenotype (Cohen’s effect size d=1.40, p<0.001). The decline of ankle-rotation velocity determined the presence of Exhaustion phenotype (d=0.98, p=0.003). The ankle-rotation power generated during the test determined the presence of Weakness phenotype (d=1.38, p<0.001). The ankle-velocity variability determined the presence of Inactivity phenotype (d=0.90, p<0.001). This study demonstrated the feasibility and validity of the iTMT to quantify frailty phenotypes. This new platform is time-efficient and doesn’t require walking test. It’s more practical for routine assessment in small and busy clinics among patients with mobility limitation.

